# High‐Performance and Stable Semi‐Transparent Perovskite Solar Cells through Composition Engineering

**DOI:** 10.1002/advs.202201487

**Published:** 2022-05-26

**Authors:** Jae Choul Yu, Bin Li, Christopher J. Dunn, Junlin Yan, Benjamin T. Diroll, Anthony S. R. Chesman, Jacek J. Jasieniak

**Affiliations:** ^1^ ARC Centre of Excellence in Exciton Science Department of Materials Science and Engineering Monash University Clayton Victoria 3800 Australia; ^2^ CSIRO Manufacturing Research Way Clayton Victoria 3168 Australia; ^3^ Center for Nanoscale Materials Argonne National Laboratory Lemont IL 60439 USA; ^4^ Melbourne Centre for Nanofabrication Clayton Victoria 3168 Australia

**Keywords:** building‐integrated photovoltaic, composition engineering, perovskite solar cells, semi‐transparent, stability

## Abstract

Semi‐transparent perovskite solar cells (ST‐PeSCs) have tremendous potential as solar windows owing to their higher efficiency and visible transmittance. However, studies toward this application are still nascent, particularly in unraveling the interplay between how the perovskite composition impacts the achievable device performance and stability. Here, the role of A‐ and X‐site modification in APbX_3_ perovskites is studied to understand their influence on these factors. Through detailed experimental and simulation work, it is found that a perovskite composition consisting of cesium (Cs) and formamidinium (FA) at the A‐site delivers the best device performance over a range of band gaps, which are tuned by changes to the X‐site anion. Using this optimized perovskite composition, power conversion efficiencies of 15.5% and 4.1% are achieved for ST‐PeSCs with average visible transmittance values between 20.7% and 52.4%, respectively. Furthermore, the CsFA‐based ST‐PeSCs show excellent long‐term stability under continuous illumination and heating. The stability of the precursor solutions across each of the studied compositions has also been considered, showing dramatic differences in the structural properties of the perovskites and their device performance for all mixed A‐site compositions possessing the archetypal methyl ammonium species, while also confirming the superior stability of the CsFA precursor solutions.

## Graphical abstract

Compositional engineering unveils that cesium and formamidinium at the A‐site deliver record efficiencies and stabilities for semi‐transparent perovskite solar cells.

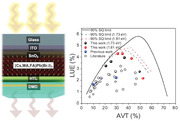

## 1 Introduction

As society strives for net‐zero carbon emissions to ameliorate the effects of anthropologically‐induced climate change, existing sources of renewable energy are being modified to expand their applicability. An example of this is solar windows, which comprise semi‐transparent solar cells (ST‐SCs) that act as an electricity source like conventional solar panels, but are also transparent enough to act as tinted windows. They have drawn significant attention in the building‐integrated photovoltaic (BIPV) market, as they greatly increase the available surface area that can be used to generate electricity in an urban environment, particularly in skyscrapers.^[^ ^1^ ^]^ Furthermore, they also have the advantage of reducing incident heat gain into buildings by partially absorbing and reflecting sunlight.

An ideal ST‐SC for solar windows must couple high photovoltaic efficiency with excellent optical properties, such as high average visible transmittance (AVT) and color rendering index (CRI) in order to give a satisfying viewer perception. While a broad range of photoabsorber materials has been considered for this application, including chalcopyrite‐, kesterite‐, CdTe‐, perovskite‐, organic‐ and dye‐sensitized‐based systems,^[^ ^2^ ^]^ the market incumbent, amorphous‐Si, has found limited application due to its relatively low efficiency, transparency, and poor aesthetic qualities.^[^ ^3^ ^]^ In response to these challenges, semi‐transparent perovskite solar cells (ST‐PeSCs) have emerged as a promising candidate for efficient semi‐transparent photovoltaics due to their superior device performance and tunable band gaps.^[^ ^2^, ^3^ ^]^

To date, many studies have focused on various fabrication methods and device designs to achieve semi‐transparency in PeSCs, such as reducing the thickness of perovskite films or fabricating island‐like perovskite films to increase transparency and color neutrality.^[^ ^4^ ^]^ However, perovskite films with these morphologies introduce practical limits to device performance in terms of reduced open‐circuit voltage (*V*_oc_) and short circuit current density (*J*_sc_) due to the increased occurrence of shunt pathways.^[^ ^4^, ^5^ ^]^ An alternative strategy for achieving high transparency ST‐PeSCs is to increase the perovskite band gap, which allows for the transmission of a broader spectrum of light below the photoabsorber band gap, thereby reducing average light absorption across the visible spectral range.

Inorganic‐organic lead halide perovskites have the chemical structure ABX_3,_ with control of the band gap afforded through changes in their constituent components.^[^ ^6^ ^]^ For a fixed B‐species, typically lead, band gap tuning has been achieved principally through X‐site halide control, with perturbations introduced by changes to the single valence cation(s) at the A site.^[^ ^7^, ^8^ ^]^ The first wide‐band gap opaque perovskite solar cells to harness these effects were based on MAPbI_3‐x_Br_x_ perovskites,^[^ ^7^ ^]^ with Yongfang Li's group reporting ST‐PeSC analogs that exhibited peak efficiency values of 11.03% at an AVT of 21% using a 200 nm‐thick MAPbI_2_Br_1_ photoabsorber layer.^[^ ^9^ ^]^ A key drawback of such MA‐based perovskites is phase separation because of the mismatch between the tetragonal phase of MAPbI_3_ and the cubic phase of MAPbBr_3_, leading to decreased device performance and stability.^[^ ^7^ ^]^ To eliminate this phase instability, Seok et al. demonstrated that incorporation of MAPbBr_3_ into FAPbI_3_ stabilized the perovskite phase.^[^ ^10^ ^]^ Later, FA was partially substituted by Cs, and structurally stable hybrid halide perovskites were obtained by tuning the halide composition in CsFAPbI_3‐x_Br_x_ formulations, resulting in stable ST‐PeSCs with an efficiency of 15.1%, albeit with no AVT reported due to the application area being tandem solar cells.^[^ ^11^ ^]^ Moreover, given that the optimal band gap of the perovskite layer in a Si/Perovskite tandem is ≈1.7 eV, most studies have focussed on optimizing for this narrow band gap range.^[^ ^12^ ^]^ As a result, for single junction ST‐SCs, the relationship between the band gap of the absorbing material and device performance and transparency has not been well explored. Therefore, it is necessary to perform a systematic study to maximize the device efficiency and stability of ST‐PeSCs, while maintaining high levels of transparency to realize the full potential of the device for solar window applications.

In this study, we investigate key factors limiting the device performance and stability of lead‐based ST‐PeSCs. First, optical simulations are used in conjunction with device measurements to elucidate the role perovskite composition and thickness play on device performance. These results provide structural parameters for optimized device fabrication. Second, we demonstrate that key to achieving high *V*_oc_ and optical transmittance is careful control of the interfacial layer between the perovskite and the hole‐transport layer (HTL) in the n‐i‐p device geometry studied. Using these optimizations for ST‐PeSCs, we achieve high power conversion efficiencies (PCEs) for CsFA‐based ST‐PeSCs between 15.4% and 4.2% with corresponding AVTs of 20.8% and 52.4%, respectively. Importantly, besides improvements in device performance and transparency, CsFA‐based ST‐PeSCs further show excellent long‐term device stability. The CsFA‐based perovskite precursor formulations are also found to exhibit superior stability over other perovskite precursor solutions, making them attractive candidates for scalable device manufacturing.

## 2 Results and Discussion

### 2.1 Compositional Dependence of Device Performance and Optical Modeling

**Figure 1:**
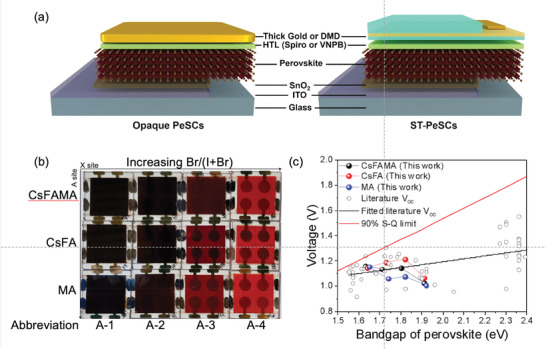
a) Schematic of the opaque and semi‐transparent (ST) perovskite solar cell (PeSC) device structures. DMD represents the semi‐transparent dilelectric‐metal‐dielectric electrode used in ST‐PeSCs. b) Photograph of perovskite devices, demonstrating color tunability through control of the cation (MA, FA, and Cs) and halide (iodide and bromide) compositions in the perovskite crystal structure. c) *V*_oc_ as a function of the perovskite band gap for perovskite devices using CsFAMA (black), CsFA (red), and MA (blue) A‐site cations. Selected *V*_oc_ values from the literature are shown as empty circles and a linear fit to these is shown by the black line.^[^ ^20^, ^22^ ^]^ The solid red line corresponds to 90% of the *V*_oc_ in the Shockley–Queisser (S–Q) limit.

In this study, we have focused on comparing compositionally modified APbI_x_Br_3‐x_ (A = CsFAMA, CsFA, and MA) perovskites prepared with similar band gaps. These have been initially assessed as neat thin films and within opaque solar cell devices (see **Figure** **1**a) to understand the optical band gap dependence and associated device performances, respectively. The exact compositions and the abbreviations used for these are included in Table S1, Supporting Information. The optical band gaps, as derived from Tauc plot analysis shown in Figure S1, Supporting Information, indicate that compositional control of the A and X sites of the perovskite thin films considered in this study afford band gap tuning between 1.63 and 1.92 eV, with the larger band gaps arising at larger Br content. The visual effects of these compositional changes on completed PeSCs are shown in Figure 1b.

The current–voltage (*J*–*V*) characteristics of these opaque PeSCs are shown in Figure S2, Supporting Information, with the extracted photovoltaic parameters summarized in Table S1, Supporting Information. Across the devices studied, those incorporating the perovskite compositions Cs_0.05_FA_0.79_MA_0.16_PbI_2.49_Br_0.51_ (CsFAMA‐1), Cs_0.17_FA_0.83_PbI_2.49_Br_0.51_ (CsFA‐1) and MAPbI_2.49_Br_0.51_ (MA‐1) had the lowest band gaps of 1.63, 1.64, and 1.65 eV, respectively. Within each respective A‐site variation these exhibited the highest PCEs of 20.2%, 20.0% and 17.0%, which is expected based on Shockley–Queisser (S–Q) limit considerations. As the Br content is increased to give the wider band gap perovskites, the photovoltaic performances of CsFAMA‐ and MA‐based compositions decrease significantly, most notably with decreases in the *FF* and *V*_oc_. In practice, a progressive decrease in the PeSC *V*_oc_ has been observed for higher band gaps, to the extent that the highest band gaps produce the lowest *V*_oc_ values for each A‐site variation,^[^ ^13^ ^]^ as seen here in Figure 1c. This loss has previously been attributed to photo‐induced halide segregation in perovskite films, with iodide‐rich phases having lower band gaps than the surrounding unsegregated phases.^[^ ^14^ ^]^ Such an adverse intrinsic material phenomenon provides practical limitations in developing optimal ST‐SCs.

### 2.2 Perovskite Film Morphology

**Figure 2:**
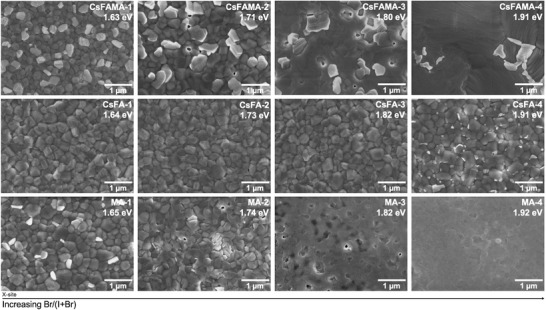
SEM images of the surfaces of perovskite films of various cation composition (CsFAMA1‐4, CsFA1‐4, and MA1‐4) and their band gaps (1.63–1.92 eV).

In order to investigate the cause of the performance reduction with increasing Br content, the morphology of the films across the various perovskite compositions was studied using scanning electron microscopy (SEM), shown in **Figure** **2**. The morphology of the MA‐based low‐bromide‐content perovskite films (MA‐1) shows compact film formation without evidence of pinholes on the surface. In contrast, the MA‐based perovskite films with high Br concentrations showed non‐uniform morphologies, with pinholes and grains of different phases and size. Previous studies have indicated that, while the presence of small amounts of MABr during the formation of MA‐based perovskites leads to an increase in the grain size, above a certain threshold, MABr causes non‐uniform perovskite crystal growth.^[^ ^9^, ^15^ ^]^ Furthermore, Jaysankar et al. reported that photoluminescence (PL) measurements of MAPbI_1.8_Br_1.2_ films (band gap 1.77 eV) showed splitting into two separate peaks under continuous illumination, which indicated segregation of the pristine MAPbI_1.8_Br_1.2_ perovskite phase into I‐rich and Br‐rich phases under illumination.^[^ ^16^ ^]^ Thus, the non‐uniform morphology and halide segregation in MA‐based perovskite films were thought to be responsible for the abrupt *V*_oc_ reduction in perovskite solar cells with band gaps exceeding 1.74 eV. Interestingly, CsFAMA‐based perovskite films demonstrated similar characteristics to their MA analogs, with film morphology degrading at higher Br concentrations. This may be due to halide segregation of a MAPbX_3_ phase in CsFAMA‐based perovskite films, the formation of which is attributed to the growth of perovskites with mixed cation and halide compositions originating from an MA‐I‐rich solvated phase and FA‐Br‐rich hexagonal phase.^[^ ^17^ ^]^ Indeed, EDS of perovskite thin films deposited here, even with a CsFAMA‐1 composition showed evidence of halide segregation (see Figure S3, Supporting Information).

In comparison, CsFA‐based perovskite films showed similar morphology regardless of halide content, as shown in Figure 2 (middle row). Similar results have been reported by many research groups using CsFA‐based wide‐band gap perovskites, with McMeekin et al. also demonstrating improved photostability (reduced halide phase segregation) at higher Br concentrations.^[^ ^11^, ^13^, ^18^ ^]^ Furthermore, our device performance and perovskite film characterization results are consistent with theoretical findings,^[^ ^18b^ ^]^ which show that exchanging the MA cation for Cs and FA cations results in better stability, likely through a combination of improved crystal stability and lower halide vacancy densities, both of which contribute to reduce the voltage loss in wide band gap perovskite solar cells.^[^ ^19^ ^]^ However, confirmation of this hypothesis requires experimental investigation of the trap state densities and recombination within the PeSCs. This validation is presented below.

### 2.3 Optical Modeling of ST‐PeSC Performance Dependence on Band Gap

**Figure 3:**
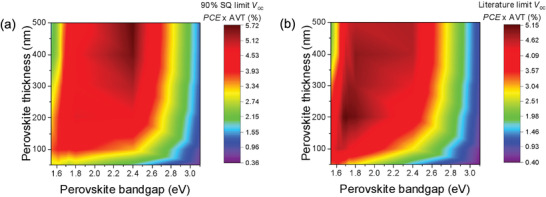
Maximum theoretical efficiency of ST‐PeSCs based on LUE (*PCE* × AVT*(%)*) with *PCE* values calculated using *V*_oc_ values of a) 90% of the *V*_oc_ in the S–Q limit and b) literature limits of the band gap‐dependent *V*_oc_.

To quantify device performance in ST‐SCs, many studies have used the light utilization efficiency (LUE = PCE × AVT), which enables a comparison between device efficiency and light transparency.^[^ ^1^, ^20^ ^]^ Consistent with this approach, in examining the theoretical limits of ST‐PeSCs here, LUE was predicted as a function of the band gap and thickness of the perovskite absorbing layer, as shown in **Figure** **3**a. The AVT and PCE values of ST‐PeSCs used as inputs for calculating these LUE values were modeled using a transfer matrix approach and assumed a voltage of 90% of the voltage derived by the S–Q limit.^[^ ^4^, ^21^ ^]^ The AVT values were calculated by averaging the total transmittance recorded between 400 and 800 nm, and did not include the photopic response in order to retain a more generalized figure of merit. Details of the optical simulations are provided in the Experimental Section. The simulations clearly show that peak LUE values are thickness dependent, with the optimal band gap increasing with increasing thickness to reach an asymptotic value of around 2.4 eV for films thickness of ≈200 nm and above. This trend reflects light transmission being both below and above the band gap for perovskite films at the lower thicknesses, which transitions to being predominantly below the band gap for thicker films.

These S–Q predictions overestimate the practical LUEs because of the band gap‐dependent *V*_oc_ losses observed for metal halide perovskites. To account for these, we collated our *V*_oc_ values with a broad range of values from literature_,_^[^ ^20^, ^22^ ^]^ and approximated their band gap dependence through a simple linear regression (Figure 1c). This regression enabled the calculation of a modified LUE that is shown in Figure 3b. It is representative of the practically achievable LUE values for current perovskite devices. As can be seen, due to the prominent voltage losses at higher band gaps, high LUE values of practical ST‐PeSCs occur across an extended band gap range between 1.7 and 2.4 eV for perovskite film thicknesses in the 100's of nanometres. Within this range, peak LUE values are predicted for practically achievable perovskite films of 100 nm thickness and above between 1.7 and 1.8 eV. For this reason, we explicitly chose materials within this narrow band gap range for further characterization and assessment within ST‐PeSCs in this work.

### 2.4 Trap State Densities and Recombination

**Figure 4:**
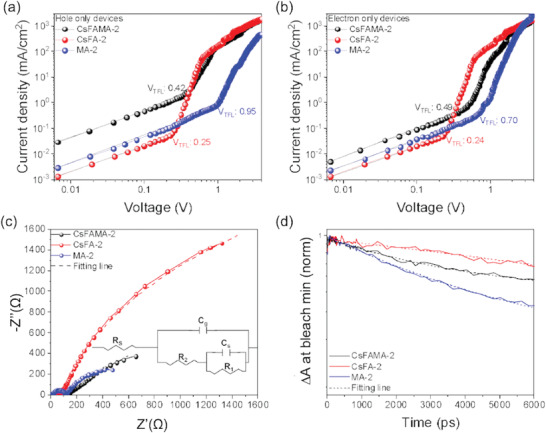
a) *J*–*V* characteristics for hole‐only (ITO/PEDOT:PSS/perovskite/VNPB/Au) and b) electron‐only devices (ITO/SnO_2_/perovskite/PCBM/Ag) with various perovskite compositions. c) Nyquist plots of ST‐PeSCs with various perovskite compositions. The inset figure shows the equivalent circuit model for PeSCs. d) Transient absorption decay probed at the band edge for various perovskite compositions.

To determine the trap‐state density in the perovskite solar cells, the *J*–*V* characteristics of electron‐ and hole‐only devices with perovskites featuring various cation compositions (CsFAMA‐2, CsFA‐2, and MA‐2) with band gaps around 1.73 eV were measured, as shown in **Figure** **4**a,b. Generally, the dark *J*–*V* characteristics can be divided into three regions: an Ohmic region, a trap‐filling limited region, and a trap‐free space‐charge limited current (SCLC) regime.^[^ ^23^ ^]^ The trap densities can be determined from the trap‐filled limit voltage (*V*_TFL_), defined by the transition between the Ohmic and trap‐filling limited regions in dark *J*–*V* characteristics. The trap density (*N*_t_) can be obtained using Equation (1):

(1) $$N_{t}=\frac{2\varepsilon_{0}\varepsilon V_{\mathrm{TFL}}}{eL^{2}}$$

where *ε*_0_ is the vacuum permittivity, *ε* is the relative dielectric constant which for perovskite is ≈35,^[^ ^24^ ^]^ L is the thickness of the perovskite film (400 nm), and *e* is the electron charge. *V*_TFL_ values for the CsFAMA‐2, CsFA‐2, and MA‐2 based devices are 0.42, 0.25, and 0.95 V in hole‐only devices and 0.49, 0.24, and 0.70 V in electron‐only devices, respectively. The corresponding hole and electron trap densities are calculated to be 1.02 × 10^16^ and 1.19 × 10^16^ cm^–3^ for CsFAMA‐2, 6.07 × 10^15^ and 5.81 × 10^15^ cm^–3^ for the CsFA‐based perovskite, and 2.30 × 10^16^ and 1.69 × 10^16^ cm^–3^ for MA‐2 based perovskites, respectively. The significantly lower trap densities in the CsFA‐based perovskite films are a clear indication of fewer defect sites. This supports the result that the *V*_oc_ and PCE of CsFA‐based perovskite solar cells is higher than for other compositions with a comparable Br content.

To further investigate the photogenerated charge recombination properties of the various perovskite compositions, impedance spectroscopy measurements of ST‐PeSCs were performed under 1‐sun illumination, as shown in Figure 4c. The equivalent circuit shown in the inset of Figure 3c was used to fit the impedance data, where *R_s_* is the series resistance, R_1_ and R_2_ represent the low‐ and high‐frequency resistances related to the recombination process at the interface of the perovskite, and the extraction, respectively, and the total recombination resistance is *R*_rec_ = *R_1_* + *R_2_*.^[^ ^25^ ^]^ *R*_rec_ values of 4184 Ω cm^2^, 7831 Ω cm^2^ and 1014 Ω cm^2^ were determined for ST‐PeSCs with CsFAMA‐2, CsFA‐2 and MA‐2‐based perovskite compositions, respectively. The higher *R_rec_* (corresponding to a lower recombination rate) of the device with CsFA‐based perovskite composition is indicative of less recombination of photogenerated charges in the perovskite layer and at the interface compared to devices with MA and CsFAMA cations. This result is consistent with the higher *V*_oc_ observed in CsFA‐2‐based solar cells compared to the other compositions with similar band gap.

To elucidate the dynamics of charge recombination occurring in the pristine CsFAMA‐2, CsFA‐2, and MA‐2 composition perovskite films, we performed transient absorption spectroscopy (TAS) measurements in the picosecond‐nanosecond time window. In Figure 4d we show the TAS dynamics probed at the peak of the band edge absorption state for each perovskite film. These datasets are well‐fitted by single exponential decays in each case. From the fitting results, CsFA‐2 perovskite films show a longer carrier lifetime, with a time constant of 16.51 (±0.63) ns, than CsFAMA‐2 and MA‐2 perovskite films with time constants of 9.18 (±0.23) and 5.40 (±0.10) ns, respectively. The shorter lifetime is attributed to faster non‐radiative recombination of charge carriers via defect or traps states in CsFAMA‐2 and MA‐2 perovskite films, which again leads to *V*_oc_ and *FF* decreases when CsFAMA‐2 and MA‐2 formulations are used in PeSCs.^[^ ^26^ ^]^ Furthermore, the CsFA‐2 perovskite film shows a longer lifetime than CsFAMA‐2 and MA‐2 perovskite films in time‐resolved photoluminescence (TRPL) measurements under excitation intensities from 2 to 70 µJ cm^‐2^ (Figure S4, Supporting Information), a result that is consistent with lower defect densities in films formed from CsFA‐based perovskite compositions. Therefore, the trap state densities, impedance, and TAS measurements all support the experimental finding that the CsFA‐2 composition has the highest device performance for a given wide band gap in these studies.

### 2.5 Performance Enhancement with Interface Passivation

Recently, 2D perovskite passivation using bulky ammonium cations has emerged as a facile approach toward achieving improved solar cell efficiencies and stabilities.^[^ ^27^ ^]^ Therefore, we have also adopted this approach by investigating the role of phenylethylammonium iodide (PEAI) and butylammonium bromide (BABr) cations as 2D perovskite surface passivation layers. These candidates have been used widely to reduce nonradiative recombination in opaque PeSCs.^[^ ^22^, ^27^ ^]^ Figure S5a, Supporting Information shows the *J*–*V* characteristics of the best‐performing 1.73 eV band gap CsFA‐2‐based ST‐PeSC (perovskite film thickness of 200 nm) without and with a 2D perovskite layer.

To evaluate the device reliability, 25 samples were fabricated and tested using various passivation layer on perovskite films. The statistics of the photovoltaic parameters are shown in Figure S5b–e, Supporting Information. These results show that the ST‐PeSCs with a BABr‐induced passivation layer exhibit higher average device performance, with the *V*_oc_ being the major characteristic to be significantly improved. Champion devices with a BABr‐induced passivation layer exhibited higher PCE (10.87%) with a higher *FF* and *V*_oc_ (but similar *J*_sc_) compared to both the device with PEAI (PCE = 10.29%) and the unpassivated reference device (PCE = 9.73%). Furthermore, the BABr treatment slightly increased the AVT of ST‐PeSCs from 35.4% to 36.7%, consistent with the minor increase in the band gap of the perovskite layer through the inclusion of additional bromide, as shown in Figure S5f, Supporting Information. The detailed device performance and AVT values of the ST‐PeSCs without and with the passivation layers are summarized in Table S2, Supporting Information. For completeness, the integrated photocurrent density derived from the external quantum efficiency (EQE) spectrum (Figure S6b, Supporting Information) of the BABr passivated ST‐PeSCs was determined to be 11.40 mA cm^‐2^, which is in good agreement with the *J*_sc_ derived from the *J*–*V* measurements. Given the success of the BABr treatment in forming the passivation layer, it was used in subsequent ST‐PeSC fabrications. Notably, the BABr passivated ST‐PeSCs were fabricated using doped Spiro‐OMeTAD as the HTL.

### 2.6 LUE, AVT, and Color of ST‐PeSCs

To confirm the accuracy of simulation results by comparing with experimental results, a series of BABr passivated ST‐PeSCs with CsFA‐2 and CsFA‐3 perovskites with band gaps of ≈1.73 and ≈1.81 eV, respectively, were fabricated in which the perovskite layer thickness was varied between 100 and 400 nm. The transmittance spectra and *J*–*V* characteristics of the completed devices are shown in Figure S7, Supporting Information and the detailed device performance and AVT values are summarized in Table S3, Supporting Information. All devices show negligible hysteresis, despite the differences in band gap and the variations in perovskite layer thickness.

**Figure 5:**
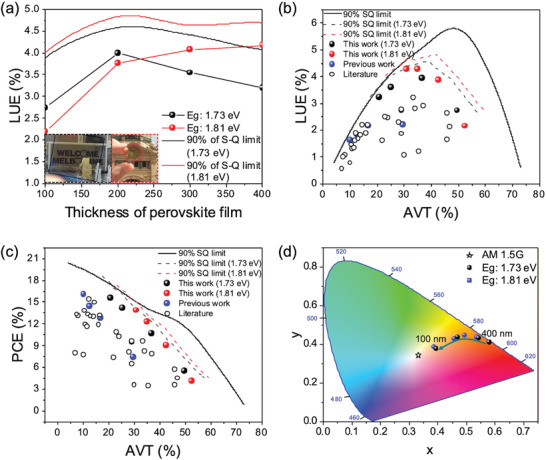
a) Dependence of LUE (PCE × AVT) values of ST‐PeSCs on perovskite film thickness. The solid lines show the predicted values based on 90% of the *V*_oc_ in the Shockley–Queisser (S–Q) limit. The inset shows photographs of the CsFA‐2 and ‐3‐based ST‐PeSCs with 100 nm thickness (with the National Gallery of Victoria (NGV) and Flinders Street Station in Melbourne in the background). b,c) LUEs and PCEs against AVTs of our ST‐PeSCs compared to our previous work and with literature (see Table S5, Supporting Information for details).^[^ ^4^, ^5^, ^28^ ^]^ All AVT values presented in this graph were determined over the 400–800 nm range. Solid lines show the maximum predicted device performance for devices operating at 90% of the performance given by the Shockley–Queisser (S–Q) limit across all band gaps. Meanwhile, the dashed line shows the predicted performance characteristics for 1.73 eV (red) and 1.81 eV (black) band gaps. d) Color coordinates on the CIE 1931 chromaticity diagram of the ST‐PeSCs with different perovskite thicknesses.

To quantify the device performance of ST‐PeSCs, we calculated LUE values of the devices, as shown in **Figure** **5**a. These results show that ST‐PeSCs with a 200 nm‐thick perovskite layer and 1.73 eV band gap exhibited an LUE value of 4.0%, while the ST‐PeSC with a 400 nm‐thick perovskite and 1.81 eV band gap had an LUE value of 4.2%. The device LUE trends match well with those predicted for the 90% of S‐Q limit, as shown in Figure 5a. Figure 5b,c further compare the LUE and PCE values, respectively, as a function of AVT of our ST‐PeSCs with those from other reports.^[^ ^4^, ^5^, ^28^ ^]^ It is evident that our optimized devices exhibit record LUE and PCEs across most AVT values reported for ST‐PeSCs in the literature to date. Notably, the asymptotic nature of both parameters at the higher AVTs reflects the need to harness higher band gap perovskites to achieve optimal performance. This is evidenced by comparing the experimental results to the maximum predicted LUE and PCE values that are independent of band gap (90% SQ limit curves).

In addition to transparency, the color of ST‐PeSCs is also a key factor for their commercialization. To quantify the characteristics of the transmitted light in ST‐PeSCs with CsFA‐based perovskite films, we calculated color‐perception indices on the CIE 1931 chromaticity diagram, as shown in Figure 5d. The corresponding color coordinates are also included in Table S4, Supporting Information. The devices with a band gap of 1.73 or 1.81 eV and a BABr‐induced passivation layer are brown in color at thicker perovskite thicknesses (400 nm), and trend toward color‐neutrality as the thickness is reduced. These results are similar to our previous work on ST‐PeSCs with CsFAMA‐based perovskite films with a band gap of 1.66 eV.^[^ ^4c^ ^]^

### 2.7 Perovskite Thin Film and Device Stability

**Figure 6:**
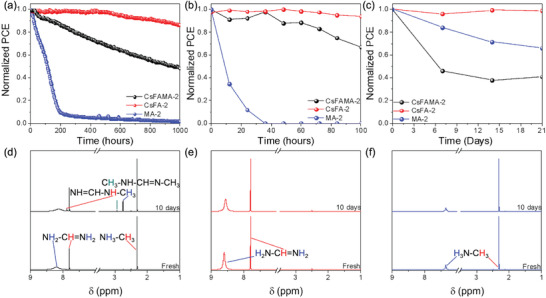
Stability characteristics of encapsulated ST‐PeSCs with various perovskite cations under a) continuous one‐sun illumination and MPP tracking and b) during thermal stability testing at 85 °C in ambient air. c) Effect of solution aging on PCE of PeSCs based on various cations. ^1^H NMR spectra of the perovskite precursor solutions when freshly prepared or aged for 10 days: d) CsFAMA‐2, e) CsFA‐2, and f) MA‐2.

To determine the long‐term stability of the ST‐PeSCs, encapsulated devices with various perovskite film compositions (APbI_2.01_Br_0.99_) and a fixed band gap of 1.73 eV were measured at maximum power point (MPP) for 1000 h of continuous 1‐sun illumination in an environmental chamber, with the results shown in **Figure** **6**a. Over this period, the MA‐2 ST‐PeSC shows significant degradation in PCE, dropping to less than 10% of its initial PCE within 200 h. The CsFAMA‐2 ST‐PeSC shows much better stability than the MA‐based device, but the performance still decreased to 50% of its initial value after 1000 h. In comparison, the CsFA‐2 ST‐PeSC shows superior stability, maintaining 85% of its initial PCE, with this stability trend in agreement with the previously reported CsFA‐based opaque devices.^[^ ^29^ ^]^

The thermal stability of the ST‐PeSCs has also been examined by measuring the device performance at 85 °C under ambient conditions over 100 h. During this time, the MA‐based ST‐PeSCs show significant degradation in PCE, dropping to less than 10% of initial PCE within 20 h. For CsFAMA‐based ST‐PeSCs, less than 70% of the original performance was maintained after 100 h. In contrast, for CsFA‐based ST‐PeSCs, nearly 100% of the initial device performance was retained after 100 h. The results reveal an enhanced thermal stability for the CsFA‐2 composition, providing evidence of the beneficial effect of the crystal stability, as predicted by previous reports (Figure 6b).^[^ ^29b^ ^]^

To further gauge these thermal stability factors, photographs and UV–vis spectra of perovskite films with various compositions (CsFAMA‐2, CsFA‐2, and MA‐2) and a thickness of 400 nm that were additionally heated at 100 °C in ambient air for 0 to 24 h are shown in Figure S8, Supporting Information. The MA‐based perovskite film rapidly decomposes to PbI_2_, with a color change to yellow after annealing for 5.5 h. For the CsFAMA‐based perovskite films, the color changes from black to red‐brown after 5.5 h, with complete decomposition observed after 24 h. In obvious contrast, the color of CsFA‐based perovskite film remains unchanged after 24 h. As can be seen in Figure S8b, Supporting Information, after being annealed for 24 h, the absorption intensity of CsFAMA‐ and MA‐based perovskite films drops dramatically in the visible range from 500 to 750 nm, whereas the absorption intensity of the CsFA‐based perovskite film shows only a slight decrease compared to the initial intensity.

Similarly, the color of perovskite films shows the same trend at an increased temperature of 150 °C in ambient air, as shown in Movie S1, Supporting Information. A significant color change in the MA‐based perovskite film starts within 6 min and all traces of color had disappeared completely from the perovskite film after 10 min. In the case of the CsFAMA‐based perovskite film, the color of the film starts to change from black to brown after 9 min and it showed complete decomposition after 20 min. In contrast, the CsFA‐based perovskite film exhibited much better stability and maintained its initial black color even after 30 min of heating at 150 °C. These results support the observed high thermal stability of CsFA‐perovskite films, which leads to an improvement in the long‐term stability of ST‐PeSCs under continuous illumination and heat conditions.

### 2.8 Impact of Precursor Solution Aging

Another key factor that can also directly impact the performance of PeSCs is the precursor solution itself, with variations giving rise to potential changes in the resultant film quality.^[^ ^30^ ^]^ As such, we compared batches of devices containing perovskite layers that had been deposited from precursor solutions of varying age, with the results shown in Figure 6c. PeSCs with perovskite films (thickness of 200 nm) prepared from fresh CsFAMA‐2, CsFA‐2 or MA‐2‐based perovskite precursor solutions exhibited PCEs of 16.4%, 15.7% and 12.6%, respectively. While the performance of PeSCs with CsFAMA‐2 remained unchanged regardless of the precursor age, both MA‐based and CsFAMA‐based perovskite films prepared from aged precursor solutions showed significantly lower PCEs due to a pronounced decrease in *J*_sc_ and *FF* (Figure S9, Supporting Information). Given the relationship between *J*_sc_ and absorbance, UV–vis spectra of films prepared from fresh and aged solutions were measured, with the results shown in Figure S10a–c, Supporting Information. These results show that the transmittance of the perovskite films is significantly changed by aging of the CsFAMA‐ and MA‐based precursor solutions, whereas the transmittance of the CsFA‐based perovskite film remains similar, even after 3 weeks of precursor solution aging, as shown in Figure S10d, Supporting Information. The decreases in *J*_sc_ for devices using aged CsFAMA‐ and MA‐based precursor solutions are consistent with these optical changes.

To clarify the influence of an aged precursor solution on the morphology of the perovskite films, we have used SEM to image the surface of the perovskite films prepared with fresh and aged precursor solutions, as shown in Figure S11, Supporting Information. The perovskite films prepared with the fresh solutions show similar morphology to that already shown in Figure 2. However, CsFAMA‐ and MA‐based perovskite films prepared using aged solutions exhibited a highly non‐uniform morphology with the formation of a rod‐like phase, which is attributed to A‐site impurities from MA decomposition products.^[^ ^31^ ^]^ In contrast, no significant change in film morphology is observed in the CsFA‐based perovskite when prepared with an aged precursor solution. These findings further support the low device performance and increased transmittance of CsFAMA‐ and MA‐based perovskite films prepared by aged precursor solutions. However, the solution stability of CsFAMA‐based perovskite precursor solutions was lower than that of MA‐based perovskite solutions, even though both yielded perovskite films with non‐uniform morphology when prepared from aged solutions.

The origins of this instability difference were probed using proton nuclear magnetic resonance spectroscopy (^1^H‐NMR), as shown in Figure 6d–f. First, CsFAMA‐2, CsFA‐2, or MA‐2‐based precursors were dissolved in deuterated dimethyl sulfoxide (DMSO‐d_6_), and then ^1^H NMR spectra were measured either within 1 h for the freshly‐prepared solution or after 10 days for an aged solution stored at room temperature in a N_2_‐filled glove box. The ^1^H signal of methyl and ammonium groups in MAI is at *δ* 2.31 ppm and at *δ* 7.36 ppm, respectively, and the ^1^H signal of methylene and amine in FAI is at *δ* 7.80 ppm and at *δ* 8.61 ppm, respectively. For the MA‐ and CsFA‐based perovskite precursor solutions, there are no obvious differences in the ^1^H‐NMR spectra between the fresh and aged solutions. In contrast, additional peaks at *δ* 2.75, *δ* 7.89, and *δ* 2.93 ppm appear in the CsFAMA‐based perovskite precursor solution due to the formation of methyl‐FAI (MFAI) and dimethyl‐FAI (DMFAI). It is proposed that the methyl group in methyl ammonium (MA) is an electron‐donating group, which enhances the nucleophilicity of the amino group, and the imine group in formamidinium (FA) is an electron‐withdrawing group and can activate the electrophilic group. This leads to an addition‐elimination reaction of the amino group in the MA and the imine group in the FA to form methyl‐ (MFA) or dimethyl‐formamidinium (DMFA).^[^ ^32^ ^]^ The chemical reactions proposed for the CsFAMA‐based perovskite precursor solution are illustrated in Figure S12, Supporting Information. These ^1^H‐NMR results explain the origins of the significant decrease in solution stability for the CsFAMA‐based perovskite precursor solution. They further confirm that the CsFA‐based perovskite composition offers improved solution and device stability compared to the other compositions given that species such as ammonium (NH_4_^+^) and dimethyl ammonium ((CH_3_)_2_NH_2_^+^), can be incorporated into perovskites as A‐site impurities with detrimental effects on the crystal structure, stoichiometry, and absorbance of the thin film.^[^ ^33^ ^]^

## 3 Conclusion

In summary, we have investigated the influence of organic‐inorganic lead halide perovskite compositions (containing blends of Cs, FA, MA, I, and Br) on the device performance and stability of semi‐transparent perovskite solar cells (ST‐PeSCs). Among these compositions, CsFA‐based perovskite devices exhibited increased device performance and stability due to reduced trap state densities and lower recombination rates of photogenerated carriers. Band gaps of 1.73 and 1.82 eV were found to be optimum for CsFA‐based ST‐PeSCs, as determined by numerical simulation and verified by SEM measurements on films and *J*–*V* characterizations of the devices. Furthermore, the effects of a perovskite surface passivation layer were investigated, with the addition of butylammonium bromide inducing the formation of a suitable passivation layer for our ST‐PeSCs, thereby resulting in an improvement in *V*_oc_ by 8% to 1.26 V. The optimized ST‐PeSCs, which were fabricated with 100–400 nm thick CsFA‐based perovskite films, yielded high PCEs of between 4.2% and 15.4% at an AVT in the 52.4–20.8% range. These CsFA‐based ST‐PeSCs also exhibited superior long‐term stability under continuous illumination, maintaining 85% of their initial PCE after 1000 h. These performance characteristics were found for devices fabricated with precursor solutions subjected to varying aging times. However, for MA‐containing perovskite precursor solutions, by‐product formation was observed, leading to decreased solution stability and quality of films formed from aged solutions. The observed benefits in precursor solution, perovskite film and device stability, and overall solar cell efficiency demonstrate the major benefits of using CsFA‐based formulations. As the expected lifetime for windows and buildings should be between 25 and 40 years, this systematic study of the perovskite composition in ST‐PeSCs is an important step forward toward realizing commercialized next‐generation solar window technologies.

## 4 Experimental Section

### Materials

Cesium iodide (CsI 99.9%), lithium bis(trifluoromethanesulfonyl)imide (Li‐TFSi 99.95%), 4‐tert‐butylpyridine (TBP, 98%), N,N‐dimethylformamide (DMF, 99.8%), dimethyl sulfoxide (DMSO, 99.9%), chlorobenzene (99.8%) were purchased from Sigma‐Aldrich. Formamidinium iodide (FAI, 99.9%), formamidinium iodide (FABr, 99.9%), methylammonium bromide (MABr, 99.9%) were purchased from Greatcell Solar. Lead iodide (PbI_2_, 99.99%) and lead bromide (PbBr_2_, 99.99%) were purchased from TCI chemicals. SnO_2_ nanoparticle solution (15 wt% in DI water) was purchased from Alfa Aesar. VNPB and Spiro‐OMeTAD were purchased from Lumtec. PEDOT:PSS (AI 4083) was purchased from Clevios Corporation. PC_60_BM was purchased from 1‐Materials.

### Fabrication of Semi‐Transparent and Opaque Perovskite Solar Cells

PeSC devices were fabricated on indium‐doped tin oxide (ITO)‐coated glass substrates (13 Ω sq^‐1^) supplied by Kintec, which were used after being cleaned sequentially by sonicating for 10 min in DI water, acetone, and IPA, drying under a stream of nitrogen gas, and then treating for 15 min under O_2_‐plasma (Harrick Plasma). A dilute SnO_2_ (3 wt% in DI water) nanoparticle solution (80 µL) was spin‐coated at 5000 rpm for 30 s onto the substrate and annealed at 150 °C for 30 min. The CsFAMA‐ and MA‐based perovskite precursor solutions were prepared by the stoichiometric mixing of the bromide (1 m of MAPbBr_3_ and of FAPbBr_3_ in 1 mL of DMF/DMSO (8:2 v/v)) and iodide (1 m of FAPbI_3_, MAPbI_3_, and CsI in 1 mL of DMF/DMSO (8:2 v/v)) stock solutions. To prepare a 1 m CsFA‐based perovskite precursor solution, CsI, FAI, FABr, PbI_2_, and PbBr_2_ powders were combined directly in the required ratio in 1 mL of the DMF/DMSO (8:2 v/v) co‐solvent blend. For control of perovskite film thickness, the concentration of the perovskite precursor solution (1.0 m for 400 nm, 0.7 m for 300 nm, 0.5 m for 200 nm, 0.25 m for 100 nm) was adjusted by appropriate dilution with the DMF/DMSO (8:2 v/v) co‐solvent blend. The prepared perovskite solutions (50 µL) were spin‐coated onto the SnO_2_‐coated ITO substrate using a two‐step method, first at 1000 rpm for 10 s, followed by 4000 rpm for 20 s. During the spin‐coating step at 4000 rpm, diethyl ether (1 mL) was deposited onto the surface after a delay of 15 s, with the substrates then annealed at 100 °C for 1 h. Where used for the surface passivation layer, PEAI or BABr solutions (0.3 wt% in IPA, 100 µL) were spin‐coated onto the perovskite layer at 5000 rpm for 30 s and the film was then heated at 100 °C for 1 min. To prepare opaque devices, a doped spiro‐OMeTAD layer was deposited onto the surface‐passivated‐perovskite layer as described in a previous report.^[^ ^4c^ ^]^ An undoped VNPB layer was deposited onto the unpassivated perovskite layer according to the previous report.^[^ ^4c^ ^]^ Subsequently, an 80 nm‐thick layer of gold was thermally evaporated under a baseline pressure of less than 8 × 10^–6^ Torr through a shadow mask. For ST‐PeSCs, a DMD (dielectric‐metal‐dielectric) electrode comprising MoO_x_ (10 nm), Au (10 nm), and MoO_x_ (35 nm) was deposited under similar evaporation conditions.^[^ ^4c^ ^]^ The active area of the opaque and semi‐transparent devices was 16 mm^2^ with four devices created on each substrate. Where described, encapsulation was applied directly after evaporation of electrodes in the glovebox using a glass coverslip and a degassed Devcon 2 Ton Clear Epoxy. To fabricate hole‐only (electron‐only) devices, PEDOT:PSS (diluted SnO_2_ nanoparticle solution at 3 wt% in DI water, for electron‐only devices) was spin‐coated onto ITO substrates at 3000 rpm for 30 s and then annealed at 150 °C for 10 min (or at 150 °C for 30 min for the SnO_2_ layer). Thereafter, a 400 nm thick layer of the relevant perovskite layer was spin coated and annealed as described above, and then VNPB at 0.2 wt% in toluene (or PC_60_BM at 2 wt% in chlorobenzene) was spin‐coated onto the perovskite layer at 3000 rpm for 30 s. Hole‐only devices were completed by the deposition of Au (80 nm) by vacuum thermal evaporation through the top electrode shadow mask as described above. Electron‐only devices used Ag (150 nm) as the top electrode, deposited as above.

### Optical Simulation

Optical simulations of transmittance of ST‐PeSCs were based on the transfer matrix method implemented in Matlab.^[^ ^4^, ^21^ ^]^ This derived the optical properties of multilayer device stacks based on the refractive indexes and thicknesses of the layers comprising the device, using (n,k) spectra for the individual layers extracted from literature as calculation inputs.^[^ ^4^, ^34^ ^]^ The layer thicknesses in the stack were determined by measurements with a Dektak 150 surface profiler (Veeco, USA). PCE values were calculated based on 90% of the *V*_oc_ in the S–Q limit and assume a value of 100% for the IQE (internal quantum efficiency). *FF* was fixed at 80% throughout for the purpose of calculating PCE.

### Characterization

The PCE of the opaque and semi‐transparent devices was determined through *J*–*V* characteristics using a VMP3 multi‐channel potentiostat (BioLogic Science Instruments) under an AM 1.5 G solar simulator provided by ABET Technologies (Sun 3000 Class AAA).^[^ ^35^ ^]^ The *J*–*V* curves measured on the direction of the scan bias at a 0.05 V s^‐1^ scan rate with 0.02 V scan step. All the *J*–*V* characteristics of the opaque and semi‐transparent devices were measured in air without encapsulation. The illuminating light intensity (100 mW cm^‐2^) was calibrated before testing using a standard silicon reference cell. Top‐surface SEM images were obtained using a Magellan 400 FEGSEM (FEI, USA) operated at 5 kV. Impedance spectroscopy was performed using an impedance analyzer (Zennium, ZAHNER‐electrik GmbH & Co.) over a frequency range from 0.5 Hz to 2 MHz under one‐sun illumination (100 mW cm^‐2^). The long‐term stability of the ST‐PeSCs was measured in a solar simulation environmental chamber (SC 340, Atlas Material Testing Tech.).^[^ ^4c^ ^]^

TAS and TRPL were performed using a 2 kHz, 35 fs Ti:sapphire laser (SpectraPhysics). For TAS, the 800 nm fundamental output was split into two branches: the first branch was frequency‐doubled to 400 nm and focused on to the sample as the pump excitation. The second was directed through a retroreflector delay stage, then focused through a sapphire crystal to generate a supercontinuum white light used as a probe of the sample transmission. The pump fluence was kept at 2 µJ cm^‐2^ to ascertain low‐fluence dynamics. Transient absorption data were generated using an Ultrafast Systems CCD detector, with mechanical chopping of the pump beam to generate pump‐on and pump‐off signals. For TRPL, the 800 nm fundamental was frequency‐doubled to 400 nm to photoexcite the samples. Photoluminescence was collected into a fiber and directed to a streak camera (Hamamatsu) for single photon counting. Both TAS and TRPL were performed on samples loaded in a nitrogen glovebox into a sealed cryostat with calcium fluoride optical windows and measured under nitrogen.

^1^H NMR spectra were recorded with a Bruker UltraShield Avance III 400 MHz Spectrometer running the TopSpin 2.1 software package at 293 K. DMSO‐d6 was used as the solvent and as an internal lock. Chemical shifts were measured in ppm.

Transmission and reflection UV–vis spectra of perovskite thin films of specified thickness and ST‐PeSCs were measured using a Perkin Elmer Lambda 1050 spectrometer fitted with an integrating sphere attachment in an ambient atmosphere. The AVT was calculated over the wavelength range of 400–800 nm.

## Conflict of Interest

The authors declare no conflict of interest.

## Supporting information

Supporting Information

Supplemental Movie 1

## Data Availability

The data that support the findings of this study are available from the corresponding author upon reasonable request.

## References

[1] C. J. Traverse , R. Pandey , M. C. Barr , R. R. Lunt , Nat. Energy 2017, 2, 849.

[2] J. Sun , J. J. Jasieniak , J. Phys. D: Appl. Phys. 2017, 50, 093001.

[3] a) Q. Xue , R. Xia , C. J. Brabec , H.‐L. Yip , Energy Environ. Sci. 2018, 11, 1688;

[4] a) E. Della Gaspera , Y. Peng , Q. Hou , L. Spiccia , U. Bach , J. J. Jasieniak , Y.‐B. Cheng , Nano Energy 2015, 13, 249;

[5] a) P. You , Z. Liu , Q. Tai , S. Liu , F. Yan , Adv. Mater. 2015, 27, 3632; 25969400 10.1002/adma.201501145

[6] M. Anaya , G. Lozano , M. E. Calvo , H. Míguez , Joule 2017, 1, 769.

[7] J. H. Noh , S. H. Im , J. H. Heo , T. N. Mandal , S. I. Seok , Nano Lett. 2013, 13, 1764. 23517331 10.1021/nl400349b

[8] a) G. E. Eperon , S. D. Stranks , C. Menelaou , M. B. Johnston , L. M. Herz , H. J. Snaith , Energy Environ. Sci. 2014, 7, 982;

[9] L. Yuan , Z. Wang , R. Duan , P. Huang , K. Zhang , Q. Chen , N. K. Allam , Y. Zhou , B. Song , Y. Li , J. Mater. Chem. A 2018, 6, 19696.

[10] N. J. Jeon , J. H. Noh , W. S. Yang , Y. C. Kim , S. Ryu , J. Seo , S. I. Seok , Nature 2015, 517, 476. 25561177 10.1038/nature14133

[11] D. P. McMeekin , G. Sadoughi , W. Rehman , G. E. Eperon , M. Saliba , M. T. Hörantner , A. Haghighirad , N. Sakai , L. Korte , B. Rech , M. B. Johnston , L. M. Herz , H. J. Snaith , Science 2016, 351, 151. 26744401 10.1126/science.aad5845

[12] A. Al‐Ashouri , E. Köhnen , B. Li , A. Magomedov , H. Hempel , P. Caprioglio , J. A. Márquez , A. B. Morales Vilches , E. Kasparavicius , J. A. Smith , N. Phung , D. Menzel , M. Grischek , L. Kegelmann , D. Skroblin , C. Gollwitzer , T. Malinauskas , M. Jošt , G. Matič , B. Rech , R. Schlatmann , M. Topič , L. Korte , A. Abate , B. Stannowski , D. Neher , M. Stolterfoht , T. Unold , V. Getautis , S. Albrecht , Science 2020, 370, 1300. 33303611 10.1126/science.abd4016

[13] a) E. T. Hoke , D. J. Slotcavage , E. R. Dohner , A. R. Bowring , H. I. Karunadasa , M. D. McGehee , Chem. Sci. 2015, 6, 613; 28706629 10.1039/c4sc03141e PMC5491962

[14] W. Mao , C. R. Hall , A. S. R. Chesman , C. Forsyth , Y.‐B. Cheng , N. W. Duffy , T. A. Smith , U. Bach , Angew. Chem., Int. Ed. 2019, 58, 2893. 10.1002/anie.201810193 30456831

[15] a) M. Yang , T. Zhang , P. Schulz , Z. Li , G. Li , D. H. Kim , N. Guo , J. J. Berry , K. Zhu , Y. Zhao , Nat. Commun. 2016, 7, 12305; 27477212 10.1038/ncomms12305 PMC4974626

[16] M. Jaysankar , B. A. L. Raul , J. Bastos , C. Burgess , C. Weijtens , M. Creatore , T. Aernouts , Y. Kuang , R. Gehlhaar , A. Hadipour , J. Poortmans , ACS Energy Lett. 2019, 4, 259.

[17] H. X. Dang , K. Wang , M. Ghasemi , M.‐C. Tang , M. De Bastiani , E. Aydin , E. Dauzon , D. Barrit , J. Peng , D.‐M. Smilgies , S. De Wolf , A. Amassian , Joule 2019, 3, 1746.

[18] a) K. A. Bush , K. Frohna , R. Prasanna , R. E. Beal , T. Leijtens , S. A. Swifter , M. D. McGehee , ACS Energy Lett. 2018, 3, 428;

[19] a) D. J. Slotcavage , H. I. Karunadasa , M. D. McGehee , ACS Energy Lett. 2016, 1, 1199;

[20] a) L. Zuo , X. Shi , W. Fu , A. K.‐Y. Jen , Adv. Mater. 2019, 31, 1901683; 10.1002/adma.201901683 31342575

[21] G. F. Burkhard , E. T. Hoke , M. D. McGehee , Adv. Mater. 2010, 22, 3293. 20517871 10.1002/adma.201000883

[22] a) T. C.‐J. Yang , P. Fiala , Q. Jeangros , C. Ballif , Joule 2018, 2, 1421;

[23] a) J. Peng , Y. Chen , K. Zheng , T. Pullerits , Z. Liang , Chem. Soc. Rev. 2017, 46, 5714; 28770935 10.1039/c6cs00942e

[24] a) B. Cao , L. Yang , S. Jiang , H. Lin , N. Wang , X. Li , J. Mater. Chem. A 2019, 7, 4960;

[25] I. Zarazúa , S. Sidhik , T. Lopéz‐Luke , D. Esparza , E. De la Rosa , J. Reyes‐Gomez , I. Mora‐Seró , G. Garcia‐Belmonte , J. Phys. Chem. Lett. 2017, 8, 6073. 29186659 10.1021/acs.jpclett.7b02848

[26] a) B. Duan , Y. Ren , Y. Xu , W. Chen , Q. Ye , Y. Huang , J. Zhu , S. Dai , Inorg. Chem. Front. 2017, 4, 473;

[27] a) Q. Jiang , Y. Zhao , X. Zhang , X. Yang , Y. Chen , Z. Chu , Q. Ye , X. Li , Z. Yin , J. You , Nat. Photonics 2019, 13, 460;

[28] a) C. O. Ramírez Quiroz , I. Levchuk , C. Bronnbauer , M. Salvador , K. Forberich , T. Heumüller , Y. Hou , P. Schweizer , E. Spiecker , C. J. Brabec , J. Mater. Chem. A 2015, 3, 24071;

[29] a) S.‐H. Turren‐Cruz , A. Hagfeldt , M. Saliba , Science 2018, 362, 449; 30309904 10.1126/science.aat3583

[30] G. S. Shin , Y. Zhang , N.‐G. Park , ACS Appl. Mater. Interfaces 2020, 12, 15167. 32176473 10.1021/acsami.9b23086

[31] A. D. Jodlowski , C. Roldán‐Carmona , G. Grancini , M. Salado , M. Ralaiarisoa , S. Ahmad , N. Koch , L. Camacho , G. de Miguel , M. K. Nazeeruddin , Nat. Energy 2017, 2, 972.

[32] X. Wang , Y. Fan , L. Wang , C. Chen , Z. Li , R. Liu , H. Meng , Z. Shao , X. Du , H. Zhang , G. Cui , S. Pang , Chem 2020, 6, 1369.

[33] J. C. Hamill , J. C. Sorli , I. Pelczer , J. Schwartz , Y.‐L. Loo , Chem. Mater. 2019, 31, 2114.

[34] a) J. Werner , G. Nogay , F. Sahli , T. C.‐J. Yang , M. Bräuninger , G. Christmann , A. Walter , B. A. Kamino , P. Fiala , P. Löper , S. Nicolay , Q. Jeangros , B. Niesen , C. Ballif , ACS Energy Lett. 2018, 3, 742;

[35] M. A. Surmiak , T. Zhang , J. Lu , K. J. Rietwyk , S. R. Raga , D. P. McMeekin , U. Bach , Sol. RRL 2020, 4, 2000097.

