# Supplementary material for: High‐Performance and Stable Semi‐Transparent Perovskite Solar Cells through Composition Engineering
Source: Adv Sci (Weinh). 2022 May 26;9(22):2201487. doi: 10.1002/advs.202201487 (PMC9353478; doi:10.1002/advs.202201487)
Supplement: Supplementary file 1 — Supporting Information [file ADVS-9-2201487-s002.pdf]

ADVANCED SCIENCE
Open Access

Supporting Information

for *Adv. Sci.*, DOI 10.1002/advs.202201487

High-Performance and Stable Semi-Transparent Perovskite Solar Cells through Composition Engineering

*Jae Choul Yu, Bin Li, Christopher J. Dunn, Junlin Yan, Benjamin T. Diroll, Anthony S. R. Chesman and Jacek J. Jasieniak**

Supporting Information

High-performance and stable semi-transparent perovskite solar cells through composition engineering

Jae Choul Yu, Bin Li, Christopher J. Dunn, Junlin Yan, Benjamin T. Diroll, Anthony S. R. Chesman, and Jacek J. Jasieniak*

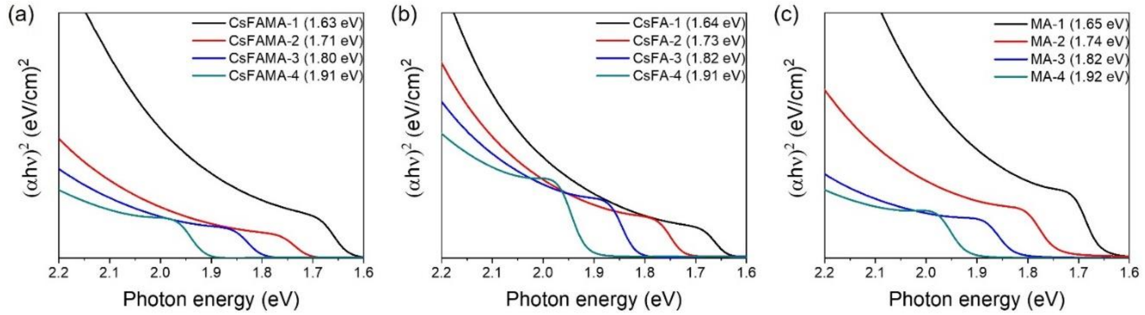

Figure S1. Tauc plots for bandgap extraction of (a) CsFAMA-, (b) CsFA- and (c) MA-based perovskite films with energy bandgaps ranging from 1.63 to 1.92 eV.

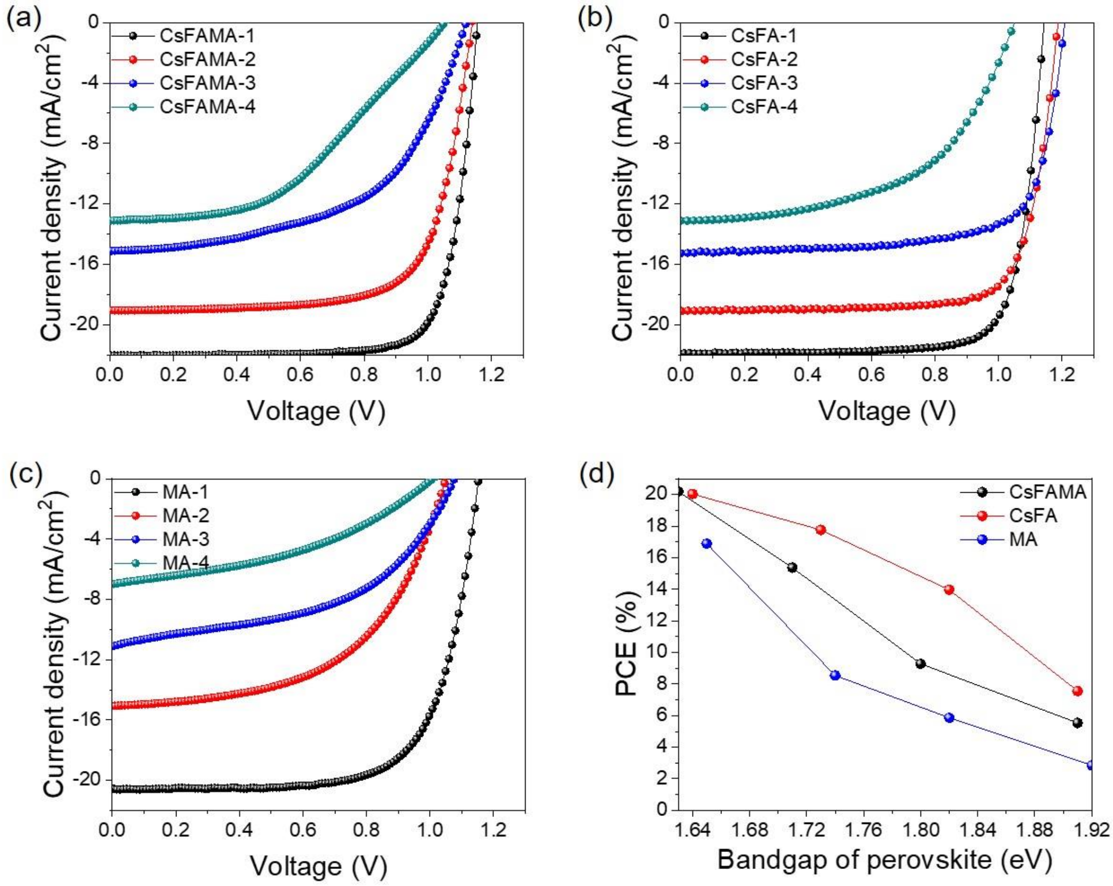

Figure S2. (a-c) *J-V* characteristics of opaque PeSCs with different perovskite compositions with band gaps ranging from 1.63 to 1.92 eV. (d) Dependence of PCE of PeSCs containing different cations on the band gap of the perovskite.

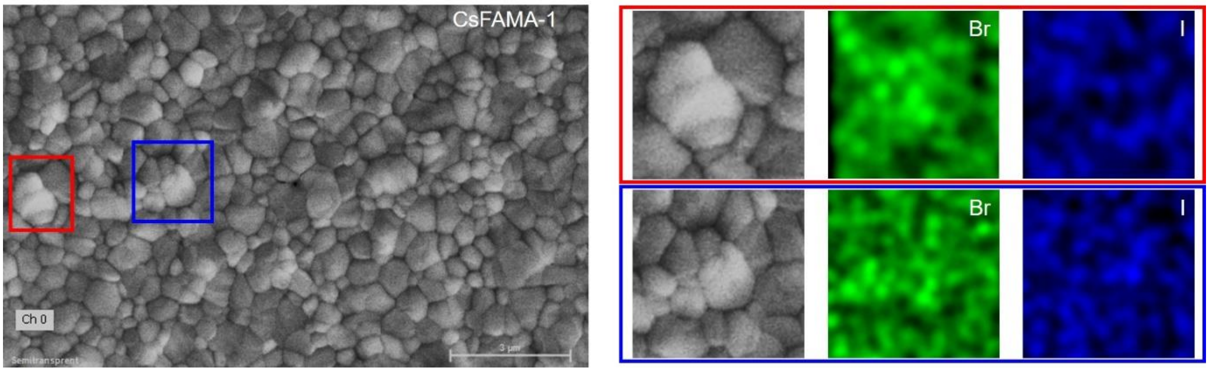

Figure S3. Top-view image and EDS mapping of CsFAMA-1 film. The red and blue boxes are enlarged SEM image of each part.

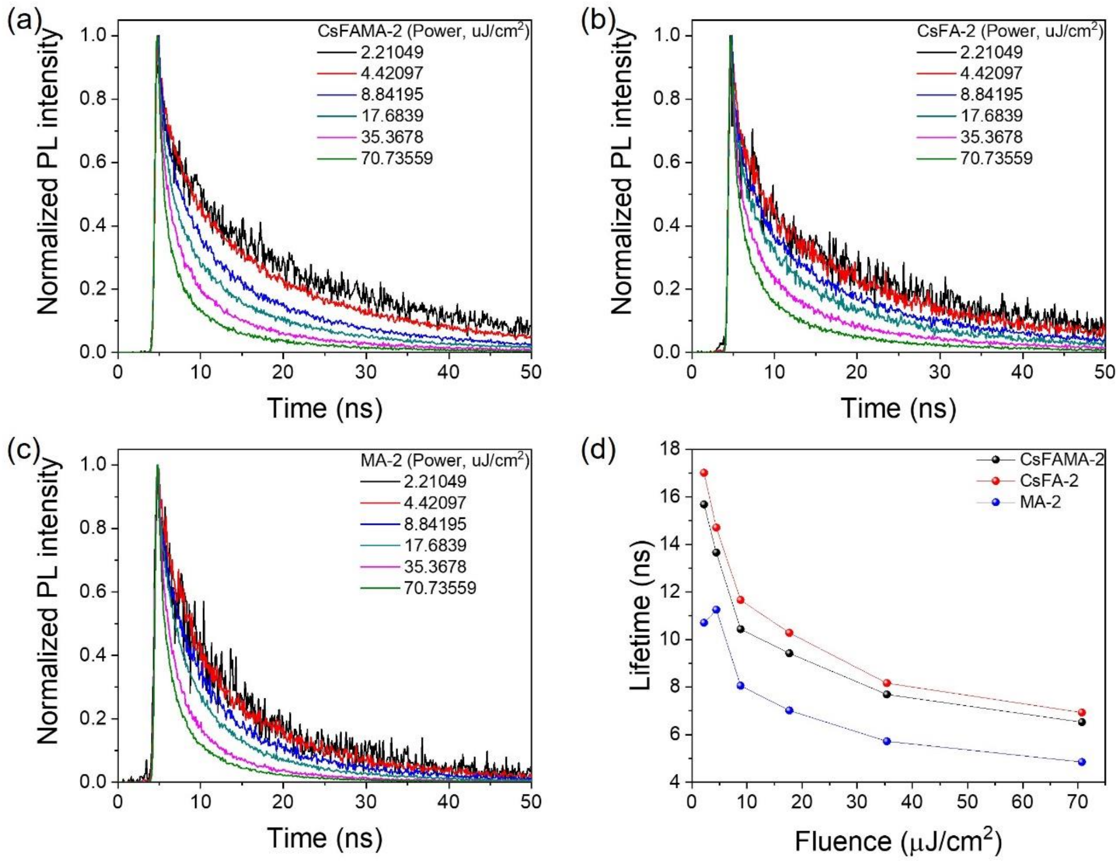

Figure S4. (a-c) TRPL spectra of CsFAMA-2, CsFA-2 and MA-2 based perovskite films at different excitation intensities. (d) Fluence-dependence of lifetimes of the various perovskite films.

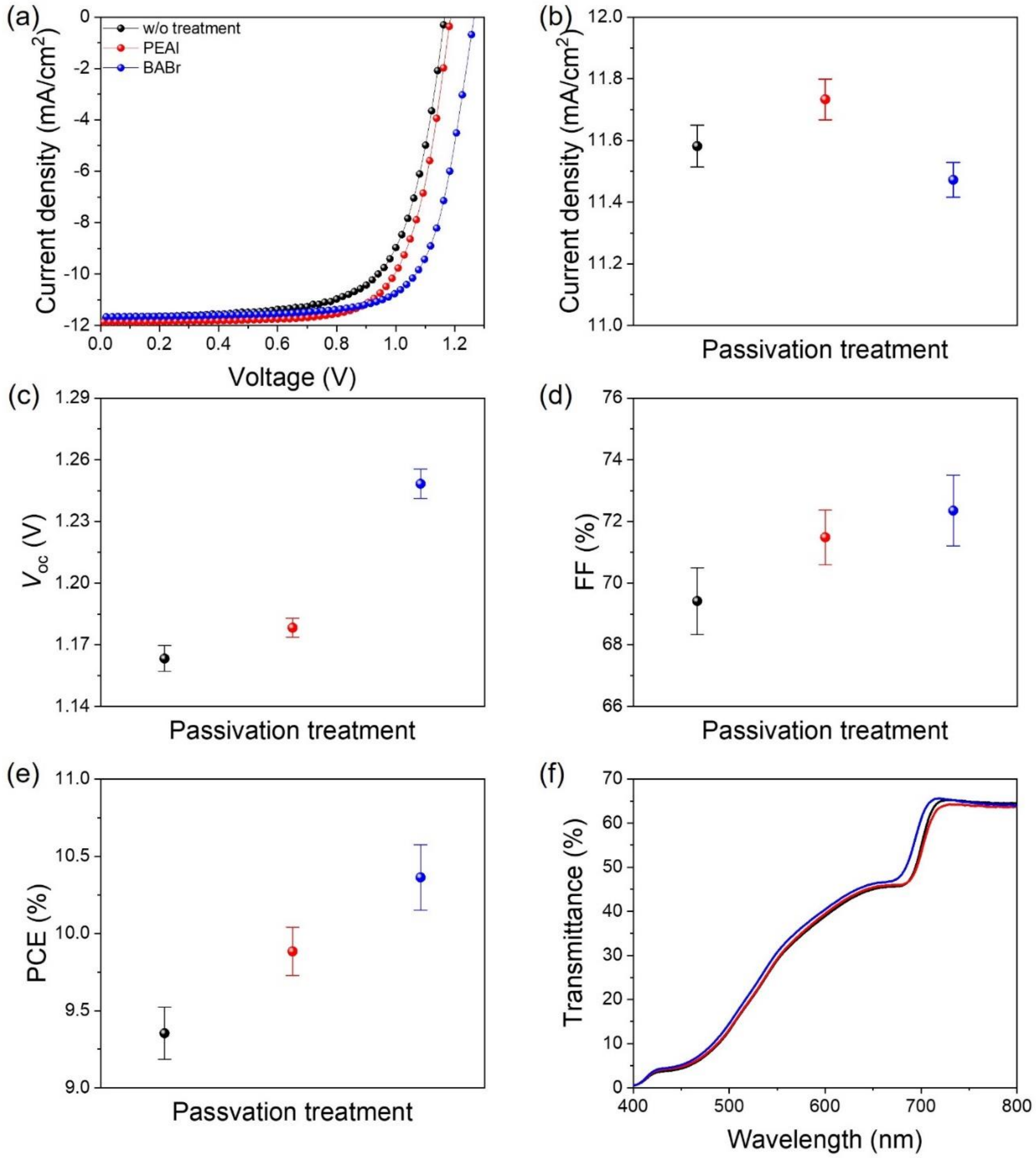

Figure S5. (a) *J-V* characteristics of best-performing and device performance statistics of ST-PeSCs based on CsFA-2 perovskite films (thickness: 200 nm) without and with PEAI and BABr treatment of the perovskite layer (b) Jsc, (c) Voc, (d) FF, and (e) PCE. (f) The optical transmittance spectra of ST-PeSCs based on CsFA-2 perovskite films (thickness: 200 nm) without and with PEAI and BABr treatment of the perovskite layer.

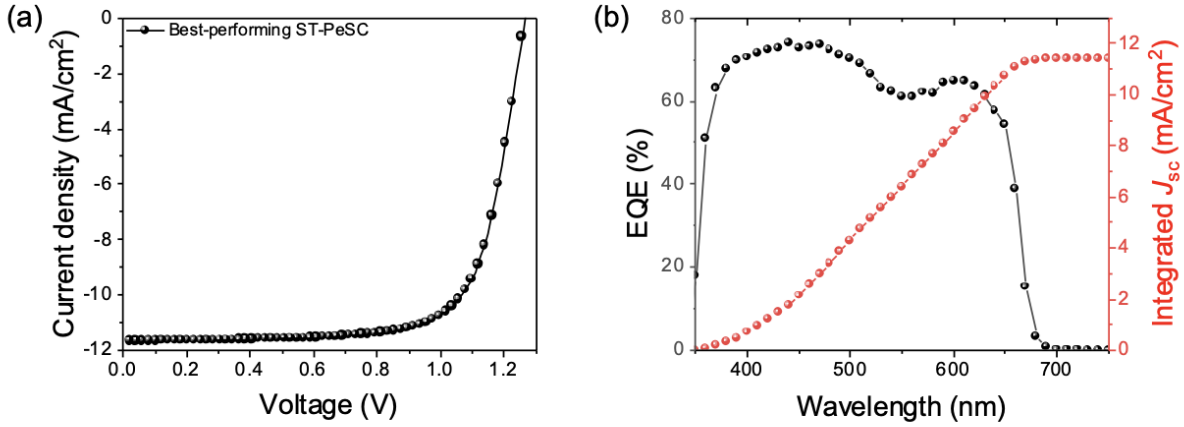

Figure S6. (a) *J*-*V* characteristics of best-performing and (b) EQE spectra of ST-PeSCs.

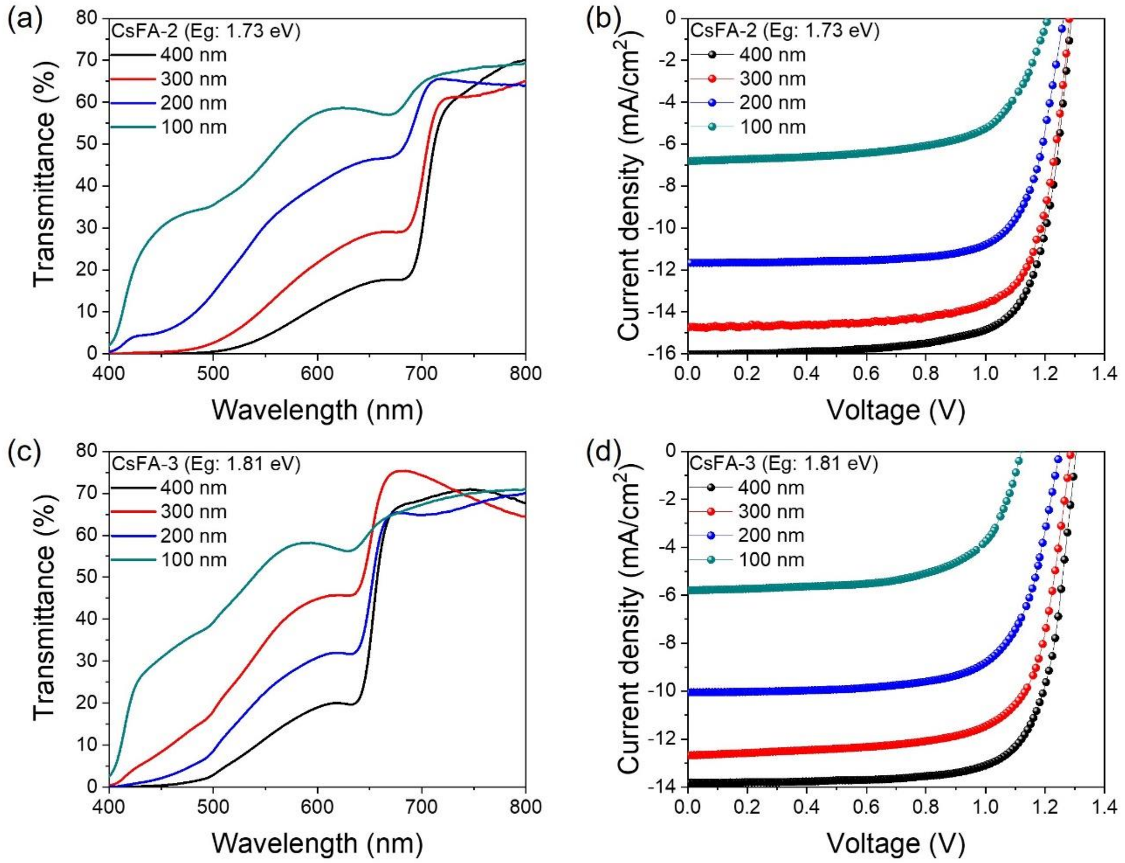

Figure S7. (a-d) Experimental transmittance spectra and *J-V* characteristics of ST-PeSCs with different thicknesses and band gaps.

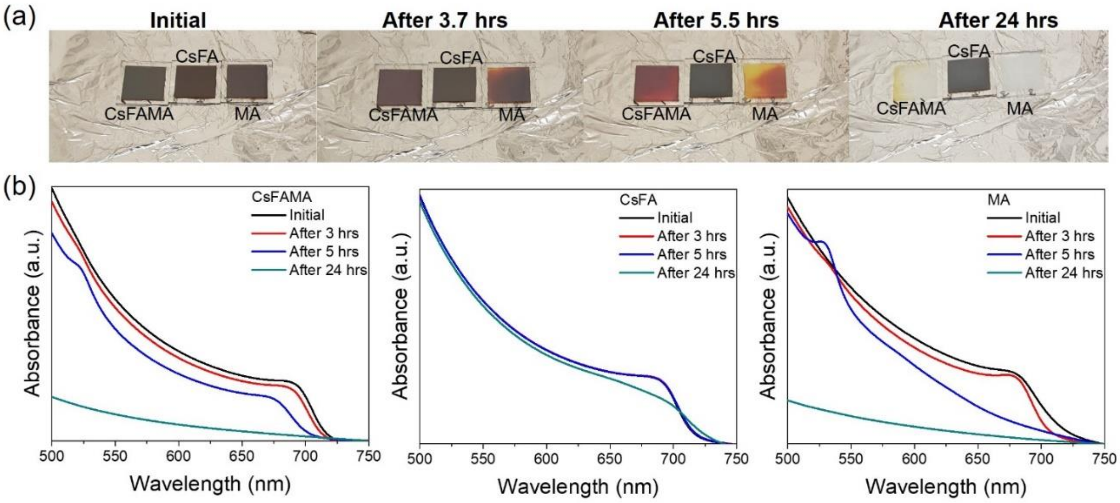

Figure S8. (a) Photographs and (b) UV-Vis absorption spectra of perovskite films fabricated with various perovskite compositions (CsFAMA-2, CsFA-2 and MA-2) annealed at 100 °C in ambient air with annealing times from 0 to 24 h.

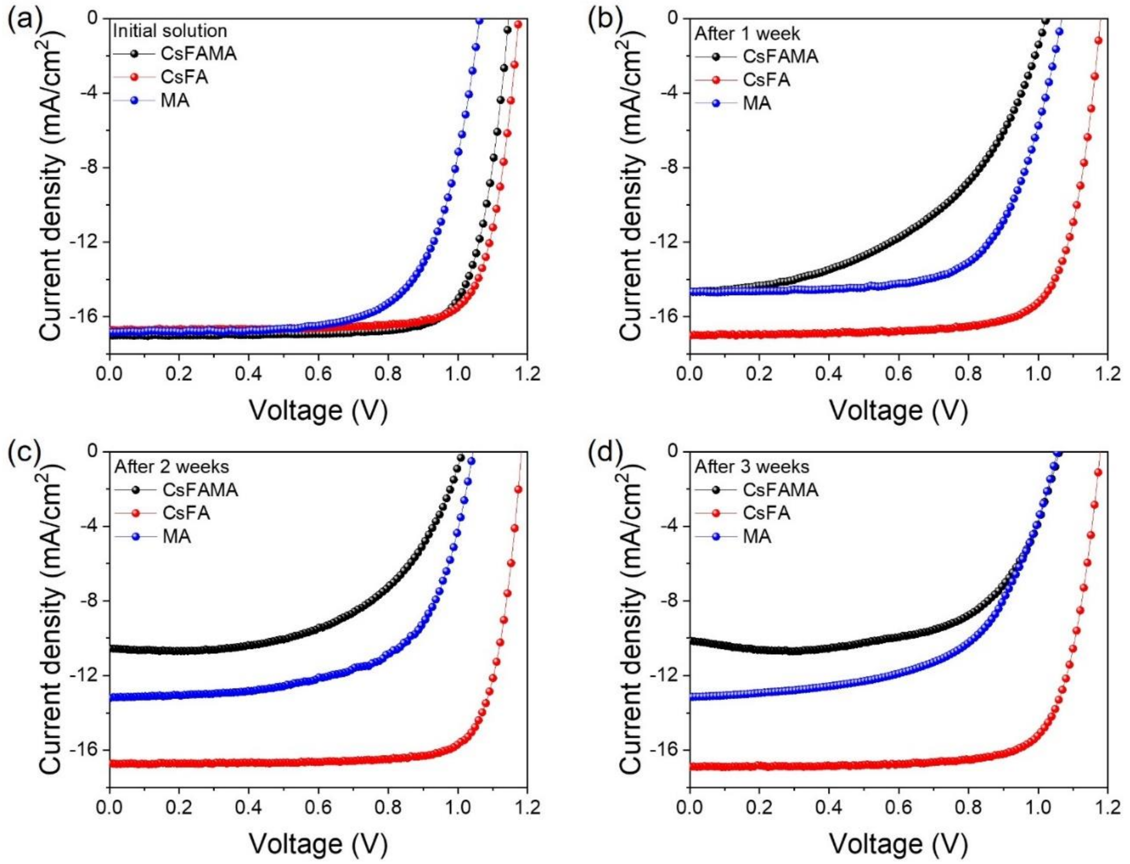

Figure S9. *J*-*V* characteristics from reverse scans of PeSCs with CsFAMA-2, CsFA-2 and MA-2 based perovskites demonstrating the effect of precursor solution aging.

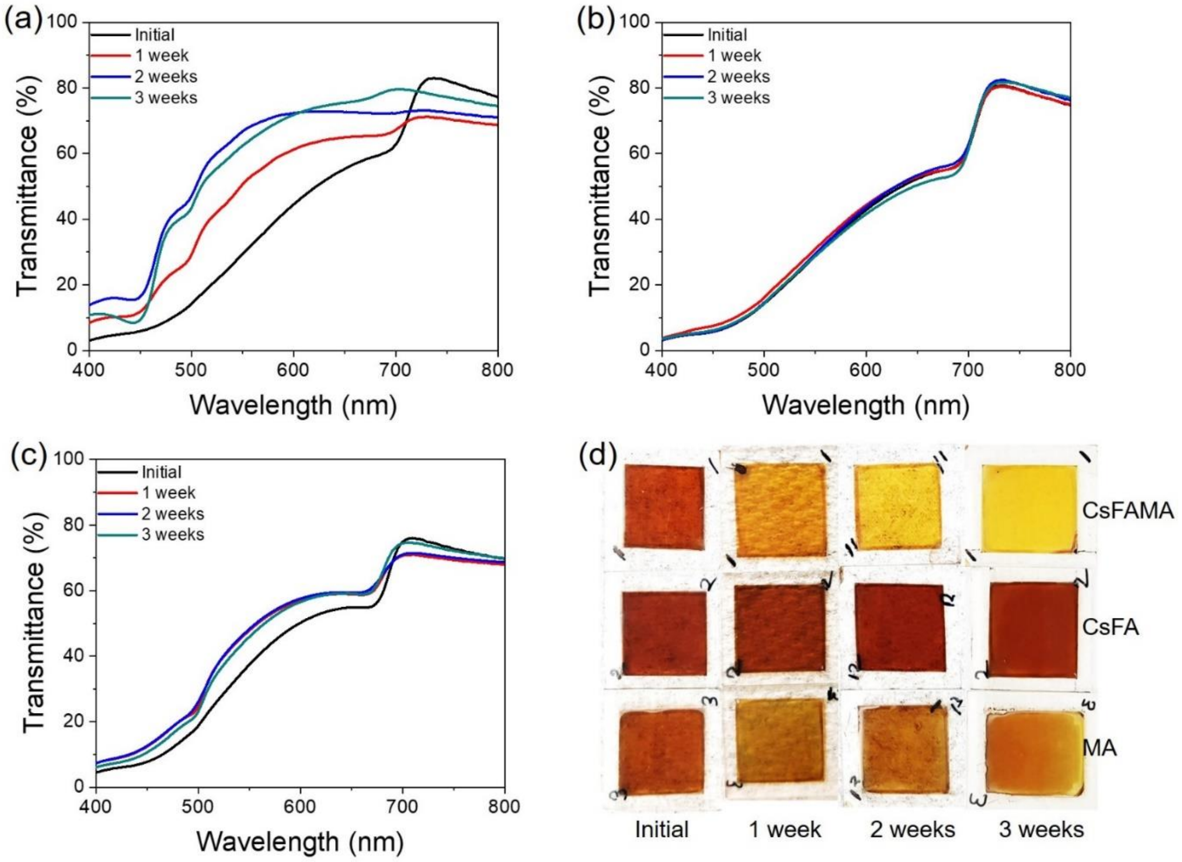

Figure S10. Effect of precursor solution aging on UV-Vis spectra of perovskite films prepared with (a) CsFAMA-2, (b) CsFA-2 and (c) MA-2 compositions. (d) Colour of perovskite films with CsFAMA-2, CsFA-2 and MA-2 compositions with different solution aging times.

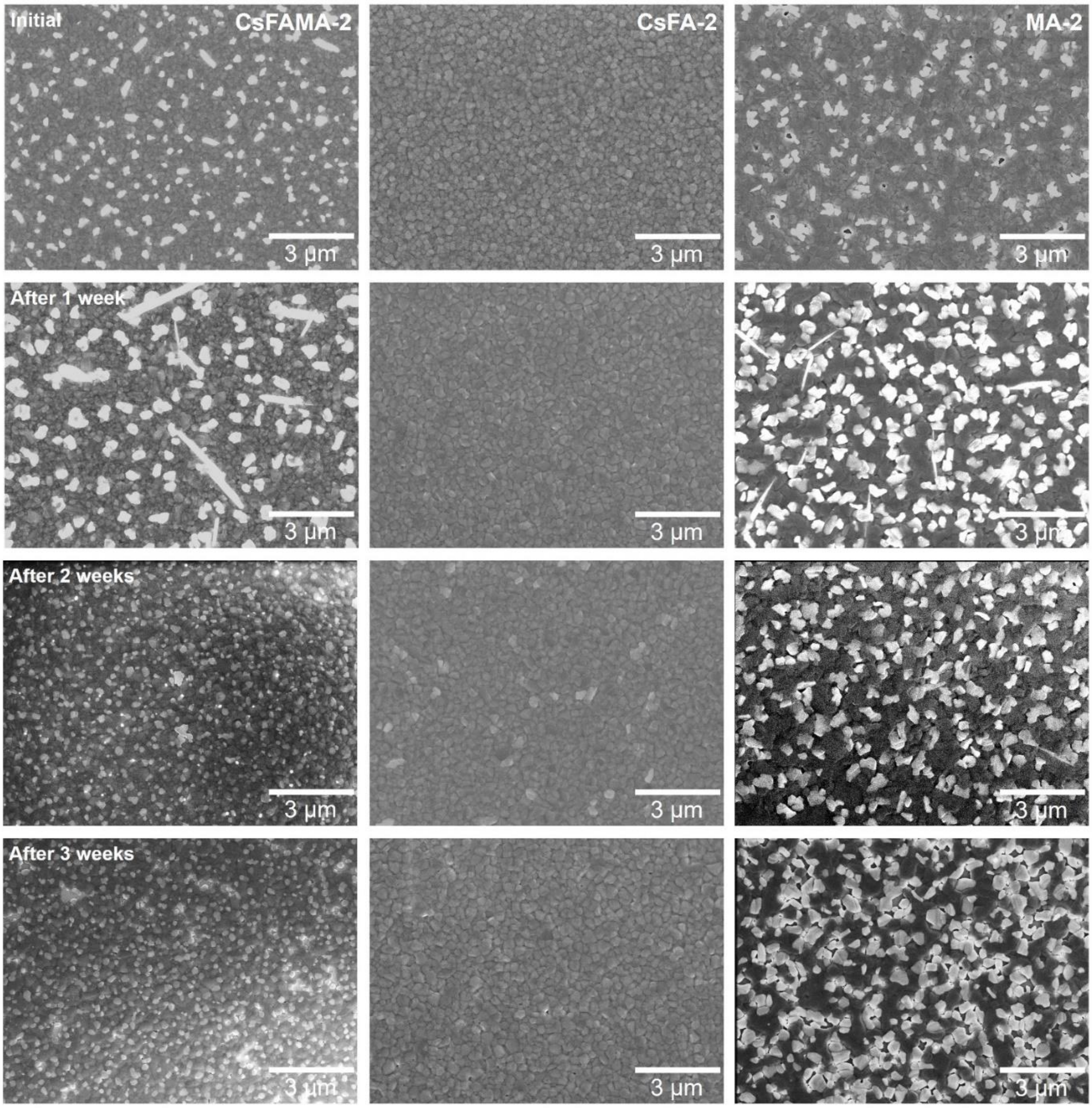

Figure S11. SEM top-view images of perovskite films of various compositions (CsFAMA-2, CsFA-2 and MA-2) demonstrating the effect of precursor solution aging.

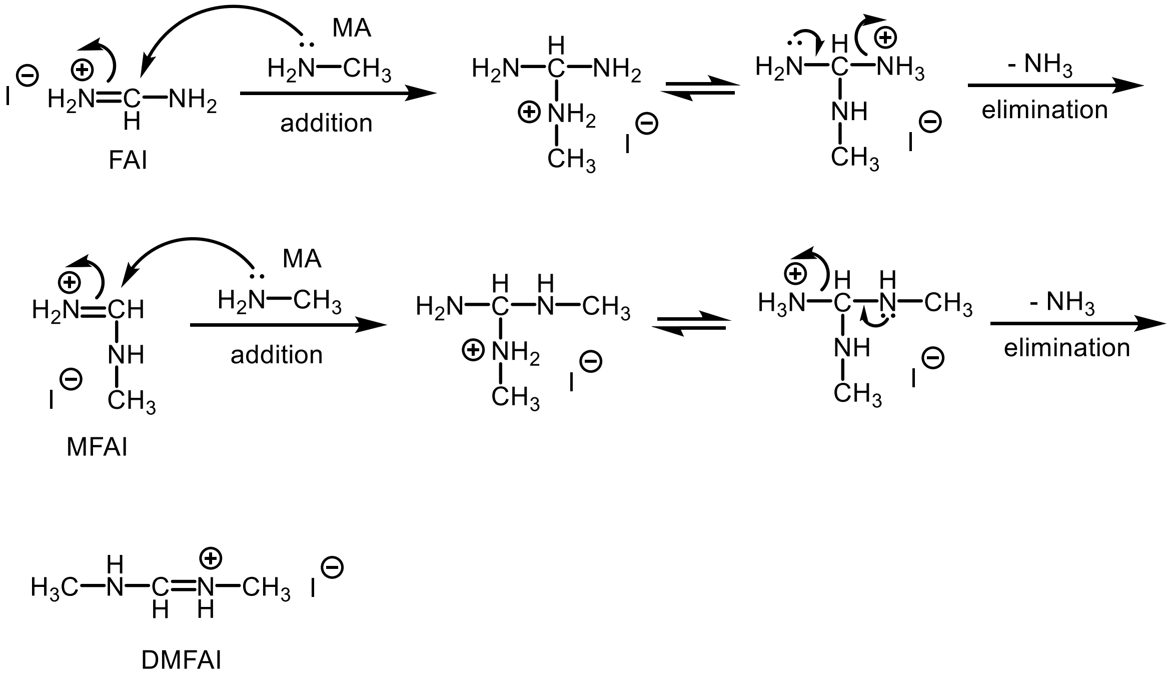

Figure S12. (h) Mechanism of possible addition-elimination reaction between MA and FA cations in the CsFAMA perovskite precursor solution.

In general, protonation of the amidine functional group N=C-N occurs on the imino nitrogen (**A**) rather than the amino nitrogen because of resonance delocalisation of the positive charge via the resonance form (**B**)

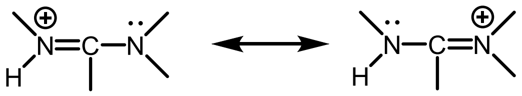

A **B**

Table S1. Summary of the device performance of opaque PeSCs with various perovskite compositions and the abbreviations used in the text.

Device configuration	Bandgap [eV]	Abbrev.	J_{sc} [mA/cm^2]	V_{oc} [V]	*FF* [%]	η [%]
CsFAMA-based PeSCs						
$Cs_{0.05}FA_{0.79}MA_{0.16}PbI_{2.49}Br_{0.51}$	1.63	CsFAMA-1	22.06	1.16	79.36	20.23
$Cs_{0.05}FA_{0.64}MA_{0.31}PbI_{2.01}Br_{0.99}$	1.71	CsFAMA-2	18.88	1.14	71.68	15.37
$Cs_{0.05}FA_{0.475}MA_{0.475}PbI_{1.5}Br_{1.5}$	1.80	CsFAMA-3	15.09	1.14	55.45	9.56
$Cs_{0.05}FA_{0.33}MA_{0.62}PbI_{1.05}Br_{1.95}$	1.91	CsFAMA-4	13.56	1.02	42.26	5.97
CsFA-based PeSCs						
$Cs_{0.17}FA_{0.83}PbI_{2.49}Br_{0.51}$	1.64	CsFA-1	21.94	1.14	79.85	20.03
$Cs_{0.25}FA_{0.75}PbI_{2.01}Br_{0.99}$	1.73	CsFA-2	19.10	1.19	78.36	17.76
$Cs_{0.25}FA_{0.75}PbI_{1.5}Br_{1.5}$ [a]	1.82	CsFA-3	15.29	1.21	75.44	13.97
$Cs_{0.25}FA_{0.75}PbI_{1.05}Br_{1.95}$ [a]	1.91	CsFA-4	13.14	1.07	54.01	7.56
MA-based PeSCs						
$MAPbI_{2.49}Br_{0.51}$	1.65	MA-1	20.63	1.15	71.61	17.05
$MAPbI_{2.01}Br_{0.99}$	1.74	MA-2	15.06	1.06	55.27	8.82
$MAPbI_{1.5}Br_{1.5}$	1.82	MA-3	10.51	1.08	50.90	5.08
$MAPbI_{1.05}Br_{1.95}$	1.92	MA-4	6.85	1.01	41.04	2.83

[a] Upper limit of Cs in CsFA composition limited to 0.25 because of low solubility of Cs component in DMF/DMSO co-solvent.

Table S2. Summary of the device performance of CsFA-2 based ST-PeSCs (200 nm perovskite thickness) without and with passivation layer.

Device configuration	J_{SC} [mA/cm^2]	V_{OC} [V]	FF	η [%]	AVT [%]
w/o treatment	11.72	1.168	71.08	9.73	35.38
with PEAI treatment	11.86	1.183	73.35	10.29	35.34
with BABr treatment	11.57	1.258	74.67	10.87	36.66

Table S3. Summary of the device performance of CsFA-2 and CsFA-3-based ST-PeSCs with different perovskite layer thicknesses under forward and reverse bias.

Perovskite formulation (bandgap) and thickness	Scan direction	J_{SC} [mA/cm^2]	V_{OC} [V]	FF	η [%]	Hysteresis index	AVT [%]
CsFA-2 (1.73 eV)							
400 nm	Forward	16.11	1.259	75.81	15.38	0.011	20.77
	Reverse	16.12	1.272	75.83	15.55		
300 nm	Forward	14.76	1.251	74.21	13.69	0.035	25.25
	Reverse	14.80	1.264	75.81	14.19		
200 nm	Forward	11.64	1.240	73.92	10.67	0.003	36.66
	Reverse	11.57	1.242	74.49	10.70		
100 nm	Forward	6.79	1.215	65.70	5.42	0.016	49.57
	Reverse	6.79	1.220	66.44	5.51		
CsFA-3 (1.81 eV)							
400 nm	Forward	13.75	1.298	74.09	13.22	0.045	30.80
	Reverse	13.88	1.302	76.67	13.85		
300 nm	Forward	12.67	1.289	73.62	12.02	0.020	34.85
	Reverse	12.70	1.289	74.95	12.27		
200 nm	Forward	9.95	1.236	69.86	8.60	0.051	42.64
	Reverse	10.09	1.250	71.82	9.06		
100 nm	Forward	5.80	1.102	64.16	4.10	0.002	52.42
	Reverse	5.77	1.125	62.99	4.09		

Table S4. Color coordinates of the completed semi-transparent solar cells.

Perovskite formulation (bandgap) and thickness	X	Y	Z	a*	b*
CsFA-2 (1.73 eV)					
400 nm	8.6	6.2	0.1	25.64	49.17
300 nm	16.6	13.4	0.7	21.83	62.55
200 nm	32.6	31.0	6.4	9.64	53.85
100 nm	48.7	47.8	27.9	7.14	22.98
CsFA-3 (1.81 eV)					
400 nm	15.7	13.0	0.8	19.25	60.43
300 nm	25.1	23.2	3.1	11.93	59.04
200 nm	36.9	35.3	9.3	9.35	49.07
100 nm	50.8	52.1	29.4	1.24	25.22

Table S5. Comparision of our ST-PeSCs with recent ST-PeSCs from the literature.

Device configuration	J_{SC} [mA/cm^2]	V_{OC} [V]	FF [%]	η [%]	AVT [%]	LUE [%]	Manuscript Reference #
ITO/PEDOT:PSS/Perovskite/PCBM/ZnO/AgNw	11.22	1.03	71.77	8.12	28	2.27	28a
FTO/TiO_2/Perovskite/Spiro-OMeTAD/Au	8.1	0.71	61	3.5	26.8	0.94	4b
ITO/SnO_2/Perovskite/Spiro-OMeTAD/MoO_3/Ag/WoO_3	17.26	1.15	66.26	13.16	16.55	2.18	28b
ITO/PEDOT:PSS/Perovskite/PCBM/C_{60}/ITO	12.1	0.95	71	8.2	34	2.79	5b
FTO/TiO_2/AAO/Perovskite/Spiro-OMeTAD/MoO_3/ITO	13.5	1.04	68.1	9.6	33.4	3.21	28c
ITO/PEDOT:PSS/Poly-TPD/Peorvskite/PCBM/Au/LiF	10.30	1.07	57.9	6.41	29	1.86	28d
ITO/TiO2/Perovskite/Spiro-OMeTAD/MoO_3/Ag/Zns	17.3	1.01	66.3	11.9	10.9	1.30	28e
FTO/TiO_2/Perovskite/Spiro-OMeTAD/PEDOT:PSS	19.2	1.11	71	15.1	8.23	1.24	28f
ITO/PTAA/Perovskite/LIF/C_{60}/PEIE/ITO	16.47	1.07	72.80	12.83	13.0	1.67	28g
FTO/SnO_2/C_{60}-SAM/Perovskite/VNPB/MoO_3/Au/MoO_3	11.78	1.01	62.3	7.41	29.54	2.19	4c
ITO/SnO_2/Perovskite/Spiro-OMeTAD/BABr/MoO_3/Au/MOO_3	12.70	1.289	74.95	12.27	34.85	4.28	This work
